# The Impact of Digital Self-Monitoring of Weight on Improving Diabetes Clinical Outcomes: Quasi-Randomized Study

**DOI:** 10.2196/54940

**Published:** 2024-04-02

**Authors:** ‪Yifat Fundoiano-Hershcovitz, Marilyn D Ritholz, David L Horwitz, Ephraim Behar, Omar Manejwala, Pavel Goldstein

**Affiliations:** 1 Dario Health Caesarea Israel; 2 Joslin Diabetes Center Harvard Medical School Boston, MA United States; 3 DLH Biomedical Consulting Las Vegas, NV United States; 4 School of Public Health University of Haifa Haifa Israel

**Keywords:** obesity, diabetes management, weight monitoring, digital health platform, self-monitoring, clinical outcome, type 2 diabetes, weight changes, blood glucose, patient empowerment

## Abstract

**Background:**

The management of type 2 diabetes (T2D) and obesity, particularly in the context of self-monitoring, remains a critical challenge in health care. As nearly 80% to 90% of patients with T2D have overweight or obesity, there is a compelling need for interventions that can effectively manage both conditions simultaneously. One of the goals in managing chronic conditions is to increase awareness and generate behavioral change to improve outcomes in diabetes and related comorbidities, such as overweight or obesity. There is a lack of real-life evidence to test the impact of self-monitoring of weight on glycemic outcomes and its underlying mechanisms.

**Objective:**

This study aims to assess the efficacy of digital self-monitoring of weight on blood glucose (BG) levels during diabetes management, investigating whether the weight changes may drive glucose fluctuations.

**Methods:**

In this retrospective, real-world quasi-randomized study, 50% of the individuals who regularly used the weight monitoring (WM) feature were propensity score matched with 50% of the users who did not use the weight monitoring feature (NWM) based on demographic and clinical characteristics. All the patients were diagnosed with T2D and tracked their BG levels. We analyzed monthly aggregated data 6 months before and after starting their weight monitoring. A piecewise mixed model was used for analyzing the time trajectories of BG and weight as well as exploring the disaggregation effect of between- and within-patient lagged effects of weight on BG.

**Results:**

The WM group exhibited a significant reduction in BG levels post intervention (*P*<.001), whereas the nonmonitoring group showed no significant changes (*P*=.59), and both groups showed no differences in BG pattern before the intervention (*P*=.59). Furthermore, the WM group achieved a meaningful decrease in BMI (*P*<.001). Finally, both within-patient (*P*<.001) and between-patient (*P*=.008) weight variability was positively associated with BG levels. However, 1-month lagged back BMI was not associated with BG levels (*P*=.36).

**Conclusions:**

This study highlights the substantial benefits of self-monitoring of weight in managing BG levels in patients with diabetes, facilitated by a digital health platform, and advocates for the integration of digital self-monitoring tools in chronic disease management. We also provide initial evidence of testing the underlying mechanisms associated with BG management, underscoring the potential role of patient empowerment.

## Introduction

### Background

People with type 2 diabetes (T2D) face challenging self-management regimens to improve glycemia and decrease morbidity and mortality while often dealing with high costs of care [1]. Obesity is one of the most common, serious, and costly medical condition in the United States, with a prevalence of 41.9% from 2017 to 2020 [2]. After a dramatic increase in its prevalence over several decades, obesity has become a major public health crisis in the United States [3]. Obesity has become one of the leading causes of death, as it is known to be the main risk factor for several noncommunicable diseases, particularly T2D [4]. It is crucial to take effective and decisive actions to hinder both the rise in the prevalence of obesity and the prevention and treatment of obesity and other obesity-related comorbidities. Approximately 80% to 90% of patients with T2D have overweight or obesity, which imposes a considerable burden on individuals, families, communities, and the health system [5,6].

Obesity and overweight are considered the primary *accelerators* for the T2D inflammatory component inducing progressive loss of beta cell insulin secretion with coexisting insulin resistance [7-9]. In addition, the expansion of white adipose tissue is related to a changed microenvironment in obesity, which impairs insulin signaling, reduces insulin-stimulated glucose transport activity, and accelerates beta cell dysfunction [10].

Previous studies have shown the beneficial effect of weight-lowering treatment on diabetes outcomes [11].

Healthful weight reduction in patients with obesity can improve glucose metabolism [12]. Weight reduction via carbohydrate-restricted nutritional intervention in patients with preobesity or obesity and prediabetes or T2D may contribute to improvement or remission in diabetes mellitus [13].

Antiobesity therapies for the treatment of patients with obesity and T2D include those that reduce body weight and improve glucose levels and other metabolic parameters. Considering the prevalence of obesity-related conditions such as adiposopathy and the fact that a significant portion of patients in cardiovascular outcomes trials for T2D had overweight or obesity, there is support for the “treat obesity first” therapeutic approach [13]. It is recommended in the guidelines for obesity that appropriate 5% to 10% weight loss can achieve significant metabolic improvement [14]. For the prevention of T2D, even modest weight reduction as little as 5%, can significantly reduce diabetes-associated complications [15,16]. Previous studies have shown that changes in various indexes such as blood lipid, blood glucose (BG), and insulin improved when weight loss reached 15% [14]. Furthermore, long-term tight weight control resulted in significant glycemic improvement, particularly demonstrated in the overweight population with T2D [17,18].

One of the goals of chronic condition management is to increase awareness and generate behavioral change to improve clinical outcomes. Behavior change for effective self-management was proven to improve health outcomes and quality of life in people living with chronic conditions such as obesity, T2D, and heart disease [19]. Underlying well-intentioned lifestyle messages is the assumption that if people deem health important, are aware of exercise and nutrition guidelines, and have access to healthy options to maintain proper levels of nutrition, diet, and exercise, then they will make healthier choices [20]. The American Diabetes Association guidelines state that lifestyle management should be intensive and involve frequent follow-ups [21].

Despite these recommendations, data from the National Health and Nutrition Examination Survey indicate that only 54.6% of patients reported receiving any diabetes education and only 13.4% had received an educational visit of any kind [16]. Earlier studies showed that helping participants with goal setting and self-monitoring of behavior, for instance, using a logbook and receiving feedback on the outcome of behavior, was associated with better intervention effects [22].

Facilitating behavior change involves using a series of strategies aimed at empowering patients, enabling them to take increasing control of their condition. This includes setting clear, achievable, and personalized goals, as well as enhancing self-efficacy [23-25]. The timing of health information and feedback focuses on when health behavior messages are delivered to people with diabetes. As diabetes care visits usually take place every 3 months, there can be a significant gap between these appointments and the daily engagement in desired behaviors. This gap makes it challenging to offer timely behavioral prompts or reinforcement [26].

In fact, patients’ mindset may modulate health outcomes, including glucose levels, in patients with diabetes [27]. Indeed, increasing perceived self-monitoring would be expected to result in subsequent health benefits [28], including glucose control in diabetes [29]. Individuals possess significant psychological influence over their health [30].

Currently, traditional health care models are being revamped with digital technologies. Digital platforms have the potential to improve our ability to enhance the delivery of health care for individual patients as well as empower patients to have more control over, and make better-informed decisions about, their health. Treatment optimization through digital health could enhance users’ alertness to their health condition through real-time monitoring, leading to effective treatments that build awareness of their daily health-related behaviors and promote increased engagement with those behaviors [31-34]. Technology-driven solutions can help people with diabetes build awareness of their daily health-related behaviors and promote increased engagement with those behaviors [32-34].

Communication of test results has been shown to be highly desired by people who have overweight, and lifestyle-focused educational messages providing advice, motivational reminders, and support have also been shown to be effective in improving chronic conditions [35]. Using a mobile platform for self-management purposes could facilitate individuals with chronic conditions in gaining insight into and controlling their BG and weight levels. Self-monitoring is a core component of behavioral obesity treatment; however, it is unknown how digital health has been used for self-monitoring and what engagement rates are achieved in these interventions [36].

Mobile apps have been shown to improve diabetes outcomes via education and support for adherence to evidence-based recommendations [37-40]. Mobile technology has emerged as a potentially useful platform to facilitate weight management [41]. Mobile apps for weight management typically offer similar features, including self-monitoring of diet and physical activity. Users can set goals within specified time frames and input data into the app, often receiving reminders or text messages. These apps have shown promising results [41]. Numerous digital health technologies have been developed to support the self-management of single chronic diseases, primarily diabetes. These technologies provide timely feedback, enhance patient education, and support the behavioral changes necessary for effective weight management. Recent research has indicated that digital self-monitoring tools can significantly influence health behaviors in patients with T2D, leading to better management of their condition [42,43]. However, given the rise in the number of people managing multiple chronic conditions, it is imperative to design and implement digital health technologies to deal with the additional complexities of multiple chronic conditions, such as the management of multiple symptoms and self-management tasks, avoiding further burden or inconvenience to the user [44-46]. Integrating the management of multiple conditions onto a single platform, where users can monitor their measurements and relevant lifestyle parameters, interact with all their data, share their data, and receive educational support, could help to minimize the known burden of multimorbidity self-management [47-49].

However, there is limited research on platforms that have been implemented to tackle multimorbidity or evaluated over longitudinal periods [45]. Specifically, the current literature is missing rigorous real-life studies to test the role of a simple self-monitoring of weight and diabetes management platform to better understand the direct association between weight monitoring and glycemic outcomes. Mainly, data are lacking on whether more frequent self-monitoring of those 2 conditions (weight and glycemia) has any impact on body weight and glycemic control in real-world clinical practice among patients with T2D and obesity [50]. In addition, many of these exclusively weight loss programs are time consuming and costly [51].

### Objectives

Our study seeks to address this gap by exploring the efficacy of digital self-monitoring of weight in managing BG levels in patients with T2D who are also managing their weight. We used a retrospective analysis of a home-use digital platform containing a diabetes BG meter and weight monitoring system with full longitudinal data capture using a supportive mobile platform among people with T2D and overweight levels. We followed users for 6 months before and 6 months after using the app for self-monitoring of weight and compared them with a matched control group that never used weight monitoring on the platform. We hypothesized that self-monitoring of weight would result in a significant improvement in BG levels. Moreover, weight monitoring (WM) will be associated with a reduction in weight levels. We also hypothesized a linkage between the changes in weight levels and the reduction in BG levels.

## Methods

### Platform

**Figure 1:**
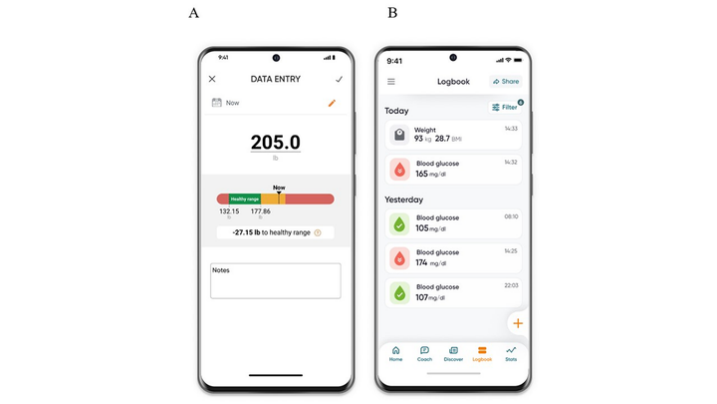
Dario mobile app platform: (A) the data entry screen allows adding the weight and height measurements to monitor where it falls within the BMI range and (B) the logbook screen presenting the blood glucose and weight measurements.

This study used the Dario Health digital therapeutics solution for chronic conditions to support the self-management of BG and weight levels. The platform combines an innovative meter with a phone app that is available for both Android and iOS devices. The glucose meter consists of a small pocket-sized holder for strips, a lancet, and the meter. The meter is removed from the holder and plugged directly into a cell phone, effectively converting the cell phone into the display screen for the meter. Weight level monitoring data are logged manually into the app on a special data entry screen (Figure 1).

Connecting the BG meter directly to the phone and adding weight levels improves the quality of data collection. Additional information for weight measurement includes an informative color scale of weight ranges reflecting the Centers for Disease Control and Prevention definitions for BMI (kg/m^2^) interpretation: <18.5 is underweight, 18.5 to 24.9 is healthy weight, 25.0 to 29.9 is overweight, and ≥30.0 is obese [52]. All information is stored in the users’ logbook in the app “attached” to the specific BG or weight reading. Data are uploaded to the cloud for backup and further analysis. Digital platform functions include interface design elements as well as specific educational content, wording, or digital interventions that affect the users’ choices in the digital environment; these functions provide personal health information and prompt feedback.

### Measures

The monthly average weight level, which was defined as the means of all of a user’s weight measurements taken over a 30-day interval, was used as the core outcome metric. The monthly average BG level, which was defined as the meaning of all of a user’s BG measurements taken over a 30-day interval, was used as another outcome metric. The mobile platform collected the following medical and sociodemographic information (by self-report) for each user: sex, age, BMI during registration, physical activity level, stress level (0=no stress and 10=very stressed), alcohol consumption (number of drinks per week), smoking (0=never and 3=yes), and added comorbidities (such as high lipids, chronic kidney disease, cardiovascular disease, sleep disorder, cancer, or stress and depression). Socioeconomic status (median household income) was matched by applying zip code data to Census [53] and app engagement (number of app sessions per month). All data were transferred and stored in compliance with the Health Insurance Portability and Accountability Act requirements using Amazon Web Services database solutions. All data were anonymized before extraction for this study.

### Study Population

A retrospective data study was performed on the Dario database on individuals who used the Dario platform between 2017 and 2023. The users purchased the device via a direct-to-consumer channel. The inclusion criteria were as follows: individuals who reported in the Dario app as diagnosed with T2D with a BG level of >140 mg/100 mL and BMI >25 kg/m^2^ in their first month on the platform (baseline) and weight monitoring system (WM group) and used the weight monitoring system (WM monitoring). The resulting group of users was matched through the propensity scores procedure with users with similar clinical parameters but who have not been using the weight monitoring (non–weight monitoring; [NWM] group).

### Study Design

The aim of our study design was to evaluate the impact of weight monitoring on BG levels. For the WM group, it was crucial to establish a clear start point for weight monitoring to assess its effects accurately. This start point is a defined intervention onset, marking when participants began actively monitoring their weight using the digital platform.

Conversely, for the NWM group, such a start point for “nonintervention” does not inherently exist, as these participants did not engage in weight monitoring. Hence, selecting a random start point for this group was a methodological necessity. This approach ensures that any observed differences in outcomes are attributable to the act of weight monitoring itself, rather than temporal factors or external influences. Therefore, the comparison between the groups hinges on the presence or absence of weight monitoring behavior. Using this approach, we enhanced the internal validity of the study. This allowed us to isolate the effect of weight monitoring from that of other variables and assess its impact on BG levels more accurately.

### Propensity Scores: Causal Inference

Propensity score matching was used in this study to address potential confounding factors and enhance the comparability of the WM and NWM groups. The rationale behind using propensity score matching lies in its ability to reduce bias and mimic the randomization process, thereby facilitating causal inference in observational studies [54].

In originally nonrandomized studies, it is common for treatment assignment (in this case, use of the weight monitoring system) to be influenced by patient characteristics and other confounding variables. These factors may introduce bias and affect the estimation of treatment effects. Propensity score matching offers a systematic approach to account for such biases and create comparable treatment and control groups [55].

The propensity score, defined as the conditional probability of receiving the treatment given a set of observed covariates, summarizes the individual’s likelihood of being assigned to the WM group. By incorporating a comprehensive set of covariates that are potential confounders, such as age, sex, initial BG and BMI levels, smoking status, alcohol consumption, stress level, comorbidities, median household income, and platform engagement, the propensity score attempts to balance the distribution of these covariates between the WM and NWM groups.

Matching participants based on their propensity scores allows a comparison between similar individuals who only differ in terms of the treatment received. This strategy helps to reduce selection bias and confounding effects, enabling a more valid estimation of the causal effect of weight monitoring on glycemic control.

The use of propensity score matching aligns with the principle of exchangeability, as it creates groups that are comparable in terms of observed characteristics. By achieving a balance on observed covariates, the propensity score matching enhances the internal validity of the study and strengthens the plausibility of causal inference from the observed associations [56].

In this study, the propensity scores were calculated for each participant using the “matchit()” function from the R package *matchit*, which followed a nearest-neighbor approach, and the distance metric used was based on logistic regression using a 1:1 ratio between the 2 study groups [57].

**Figure 2:**
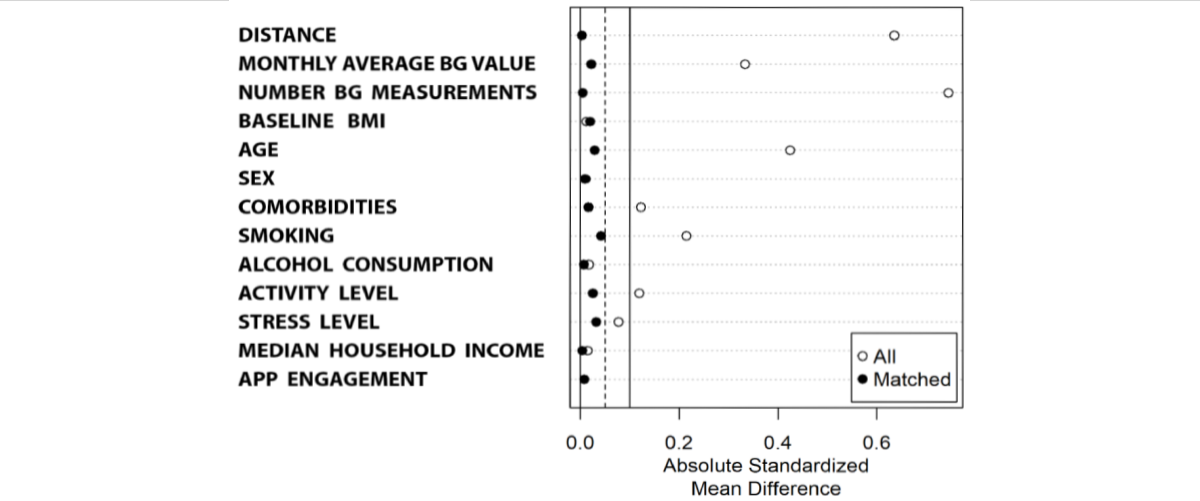
The plot presents the efficacy of the matching procedure for balancing the groups. A caliper width of 0.05 SDs was reached for all the parameters except alcohol consumption, which remained within 0.1 SDs. The variable labels are as follows: monthly average BG value=monthly average blood glucose value, number BG measurements=monthly number of blood glucose measurements, baseline BMI=starting BMI value, age=reported age, sex=reported sex of the users, comorbidities=sum of reported comorbidities (hypertension, high blood lipids, kidney disease, cardiovascular disease, sleep disorder, cancer, depression, and no comorbidity), smoking=reported smoking, alcohol consumption=reported alcohol consumption, activity level=reported level of daily activity, stress level=reported level of stress, median household income=median household income matched based on zip code, and app engagement=number of monthly app sessions. BG: blood glucose.

To achieve balanced groups, nearest-neighbor matching with a caliper width of 0.1 SDs of the propensity score was applied. The matching procedure aimed to identify, for each WM user, a corresponding NWM participant with the closest propensity score. Participants without suitable matches were excluded from the analysis. Figure 2 presents the efficacy of the matching procedure for balancing the groups. A caliper width of 0.05 SDs was reached for all the parameters except alcohol consumption which remained within 0.1 SDs.

### Ethical Considerations

All data used for the analysis were anonymized before extraction for this study. The study received an exemption from the institutional review board under the Ethical and Independent Review Services, a professional review board, which issued the institutional review board exemption for this study (18032-06#) [58]. The users who participated in the study were provided with a Terms of Use document mentioning the legally valid consent of the end user for the company to collect and access their information. The use of the app, site, or services shall be deemed to constitute user consent to be legally bound by these Terms and the Privacy Policy. Please refer to the current “Terms-of-use” at the link [59].

### Analytic Approach

Traditionally, a classical linear longitudinal model assumes a single-slope growth pattern for changes in an outcome variable over time. However, empirical data often exhibit more complex patterns that cannot be adequately captured by such a simple model. In our study, we used piecewise-based mixed-effects models to allow for greater flexibility in modeling trajectories over time [60].

The piecewise model approach offers the advantage of accommodating different linear trends in the data across distinct regions. Specifically, we used a mixed piecewise model to assess differential changes in the WM and NWM groups in the monthly average BG level in 2 segments: before and after weight monitoring use. This approach enabled us to capture the potential changes in BG trajectories associated with the introduction of the monitoring system. Using a statistical model that accounted for curvilinear changes, we were able to test the complex effects and capture the dynamics of the associated BG fluctuations.

For the analysis, the time data were centered around the beginning of the weight monitoring period. We included a 6-month timeframe before and after this point to capture the potential impact of weight monitoring use. In the NWM group, we randomly selected a cutoff point and included data collected during the 6 months before and after the simulated cutoff point. To model the temporal changes in the monthly average weight level between the WM and NWM groups, we fitted a piecewise-based mixed-effects model. The piecewise cutoff point was set at the initiation of weight monitoring, assuming a change in the time-related trajectory of the monthly average BG level between the 2 groups. We incorporated interaction terms between the time trajectories and groups to capture this differential effect. Thus, 2 time parameters (pre and postintervention) were used as covariates, the groups (WM and NWM) were considered as a factor, and the monthly number of BG measurement served as a potential confounding variable. All the tests were 2-tailed and the type 1 error was set to 5%. The model included random intercepts and random slopes for the time trajectory after the piecewise cutoff, accounting for individual variability in BG changes.

In addition, we used mixed model analysis to examine the time trajectory of BMI changes (covariate) for the initial 6 months of weight monitoring in the WM group, controlling for baseline BMI and the number of monthly BMI measures as confounding variables. These models included random intercepts and random slopes of the time trajectory to capture individual variations in weight changes over time. Unstandardized regression weights (B), test statistics (*t*), and associated significance (*P* values) were reported.

Finally, the monthly BMI levels were disaggregated to separate within- and between-person variabilities using person-level centering and person-level aggregation [61]. In addition, a 1-month lagged within-person BMI was calculated. Thereafter, a mixed model was applied to test the 1-month lagged and simultaneous association of monthly within-person BMI changes and between-person BMI with the monthly average BG level. All the model predictors were defined as covariates.

## Results

### Users

In total, 1932 users were included in the study. The WM group included 50% users, and the NWM group, matched through the propensity scores procedure, included 50% users. The study cohort comprised 51.6% (997/1932) of men, and 60.82% (1175/1932) of the participants had comorbidities. The average age of the participants was 62.8 (SD 12.5) years, with an average BMI of 35.4 (SD 7.3). The median household income for the participants was US $68,200 (SD US $25,100). The distribution of the other parameters is presented in Table 1 by study group.

No differences were found between the WM and NWM groups. The study included individuals with diabetes who monitored their BG levels and weight using the Dario platform.

The distribution of various sample characteristics overall and by WM and NWM groups is presented in Table 1, and any significant differences were shown.

**Table 1:** Distribution of various sample characteristics overall and by WM^a^ and NWM^b^ groups.

		NWM (n=966)	WM (n=966)	Total (N=1932)	*P* value	
Monthly BG^c^, mean (SD)		170 (40)	180 (41)	175 (40)	.62	
Number of BG measurements, mean (SD)		39 (33)	39 (29)	39 (31)	.92	
Baseline BMI, mean (SD)		36 (7.6)	35 (7.0)	35 (7.3)	.68	
Age (years), mean (SD)		63 (12)	63 (13)	63 (12)	.52	
**Sex, n (%)**						.86
	Male	496 (51.3)	501 (51.9)	997 (51.60)		
	Female	470 (48.7)	465 (48.1)	935 (48.39)		
Number of comorbidities, mean (SD)		1.1 (1.1)	1.1 (1.2)	1.1 (1.2)	.71	
Smoking, mean (SD)		0.25 (0.44)	0.27 (0.45)	0.26 (0.44)	.35	
Alcohol consumption, mean (SD)		1.4 (3.3)	1.3 (3.4)	1.3 (3.4)	.88	
Physical activity level, mean (SD)		4.1 (2.4)	4.2 (2.2)	4.1 (2.3)	.59	
Stress level, mean (SD)		5.1 (2.5)	5.1 (2.5)	5.1 (2.5)	.48	
Median household income, mean (SD)		68,000 (25,000)	68,000 (25,000)	68,000 (25,000)	.94	
App engagement, mean (SD)		56 (53)	56 (52)	56 (52)	.86	

^a^WM: weight monitoring.

^b^NWM: non–weight monitoring.

^c^BG: blood glucose.

### Weight Monitoring Is Associated With BG Levels

**Figure 3:**
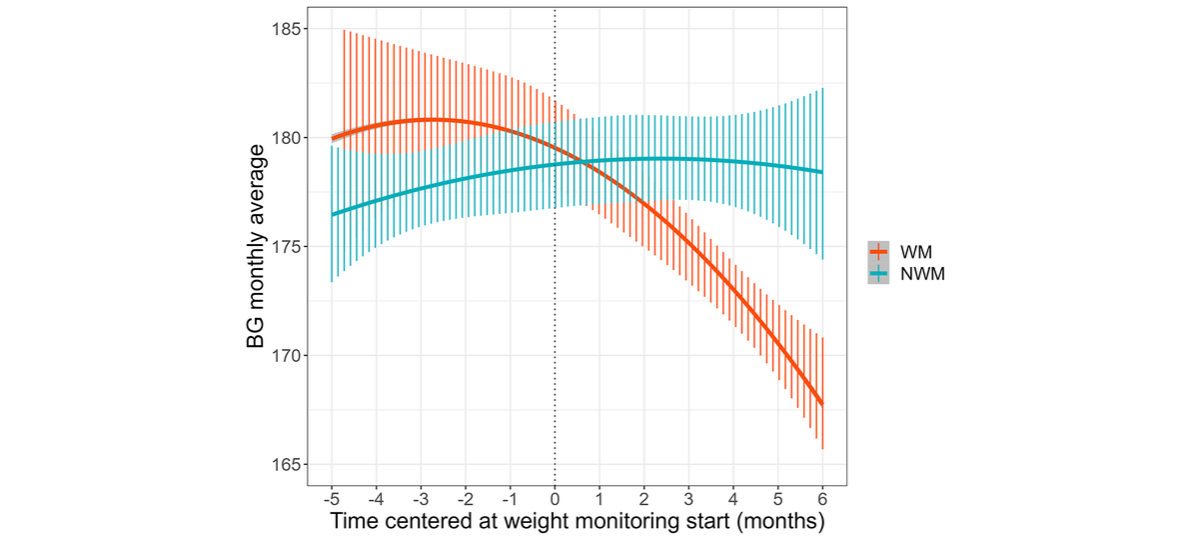
Monthly average blood glucose (BG) fluctuation for the weight monitoring (WM) group and the non-WM (NWM) group. Zero on the x-axis means the start of WM. Vertical lines represent a 95% CI over time.

The results from the piecewise mixed model analysis indicated a significant interaction between the time trajectory, starting weight monitoring and the group (B=3.02; *t*=6.03; *P*<.001) on BG levels (Table 2). Specifically, the WM group demonstrated a significant reduction in the BG levels (B=−2.81; *t*=−8.88; *P*<.001), whereas the NWM group did not exhibit a significant time trend (B=0.21; *t*=0.55; *P*=.59; Figure 3). Before weight monitoring, there was no significant difference observed in BG time trends between the 2 groups (B=0.69; *t*=1.06; *P*=.29). Furthermore, we investigated the proportion of users who achieved a BG level reduction in their last month of measurement less than the average BG levels of 154 mg/100 mL, 183 mg/100 mL, and 212 mg/100 mL, equivalent to estimated glycated hemoglobin (HbA_1c_) of 7.0, 8.0, and 9.0, respectively [62]. Remarkably, of the 966 users examined per group, 45% (435/966) versus 36% (348/966), 71% (686/966) versus 59% (570/966), and 85% (821/966) versus 76% (734/966) of the WM versus NWM individuals demonstrated substantial reductions in HbA_1c_ levels of <154 mg/100 mL, 183 mg/100 mL, and 212 mg/100 mL, respectively (*P*<.001 for all).

**Table 2:** Piecewise mixed model analysis of the weight monitoring affecting the monthly BG^a^ levels^b^.

		Outcome: monthly BG levels		
		Estimates (B, 95% CI)	Statistic *t*	*P* value
**Predictors**				
	Intercept	192.63 (189.05 to 196.20)	105.62	<.001
	#BG measurements^c^	−0.22 (−0.25 to −0.19)	−14.11	<.001
	time1^d^	−0.17 (−1.34 to 1.00)	−0.29	.77
	group (NWM^e^)	−2.19 (−6.93 to 2.55)	−0.91	.37
	time2^f^	−2.81 (−3.43 to −2.19)	−8.88	<.001
	time1×group (NWM)	0.69 (−0.58 to 1.96)	1.06	.29
	time2×group (NWM)	3.02 (2.04 to 4.00)	6.03	<.001

^a^BG: blood glucose.

^b^σ2 residual variability=843.15; τ00 UID random intercept=2099.24; τ11 UID.time2 random slope of the second slope=53.68; ρ01 UID: covariance between the random intercept and slope=−0.27; intraclass correlation=0.72.

^c^#BG measurements=number of BG measurements per month.

^d^time1represents the piecewise slopes before the weight monitoring intervention.

^e^NWM: non–weight monitoring.

^f^time2 represents the piecewise slopes after the weight monitoring intervention.

### BMI Fluctuations and the Link to BG

During the weight monitoring period (Table 3) of the WM group, a significant decrease in BMI was observed (B=−0.13; *t*=−9.35; *P*<.001).

Interestingly, the number of monthly measurements was negatively associated with BMI (B=−0.003; *t*=−2.22; *P*=.03). Furthermore, the findings of the lagged analysis disaggregating within- and between-person variabilities shed light on BMI as a potential mechanism driving BG (Table 4). Specifically, the analysis demonstrated that an increase in within-person BMI was associated with elevated BG levels (B=4.67; *t*=3.47; *P*<.001). Similarly, an increase in between-person BMI was found to be associated with higher BG levels (B=0.61; *t*=2.65; *P*=.008). However, the 1-month lagged back BMI was not associated with BG levels (B=−0.77; *t*=−0.91; *P*=.36).

**Table 3:** BMI fluctuations over time in the WM^a^ group^b^.

		Outcome: BMI		
		Estimates (B, 95% CI)	Statistic *t*	*P* value
**Predictors**				
	Intercept	0.88 (0.49 to 1.26)	4.48	<.001
	Monthly number of weight measurements	−0.006 (−0.01 to −0.002)	−2.22	.03
	Baseline BMI	0.98 (0.97 to 0.99)	180.41	<.001
	Time^c^	−0.13 (−0.16 to −0.10)	−9.35	<.001

^a^WM: weight monitoring.

^b^σ2 residual variability=0.24; τ00 UID random intercept=1.29; τ11 UID.time random slope of the time=0.17; ρ01 UID covariance between the random intercept and slope=−0.25; intraclass correlation=0.93.

^c^time represents the slope over 6 months after the intervention.

**Table 4:** Within- and between-person associations between BMI and BG^a^ in the WM^b^ group.

			BG				
			Estimates (B, 95% CI)		Statistic *t*		*P* value
**Predictors^c^**							
	Intercept	147.47 (131.49 to 163.45)		18.09		<.001	
	BMI within-person	4.67 (2.03 to 7.30)		3.47		.001	
	BMI between-person	0.61 (0.16 to 1.07)		2.65		.008	
	BMI within-person lag (−1 month)	−0.77 (−2.43 to 0.89)		−0.91		.36	

^a^BG: blood glucose.

^b^WM: weight monitoring.

^c^σ2 residual variability=464.96; τ00 UID random intercept=1684.51; τ11 UID.time random slope of the within-person BMI=325.17; ρ01 UID covariance between the random intercept and slope=−0.01; intraclass correlation=0.80.

## Discussion

### Principal Findings

This study examined the ability of people with diabetes to regulate BG levels through simple weight monitoring. It used propensity score matching for the control group and used a piecewise mixed model as a statistical framework to describe the nonlinear behavior in BG levels, comparing 2 user cohorts over time. Our analysis indicated that before the weight monitoring phase, both groups demonstrated flat trajectories in BG levels. However, after starting the use of the self-monitoring of weight, the WM group experienced a significant reduction in BG levels, whereas the NWM group’s BG levels remained flat.

In addition, by disaggregating within- and between-person BMI variabilities, we showed an association between both BMI sources and BG levels, suggesting that general BMI levels and BMI fluctuations can potentially contribute to BG modulation. However, a lagged analysis did not find an association between within-person BMI fluctuations and next-month BG levels, which does not support the claim of BMI as a potential mechanism of BG changes.

This study demonstrates that the use of digital tools for self-monitoring of weight can significantly affect BG levels in patients with T2D. This finding offers a practical approach to enhancing T2D management, especially for the majority of patients who are also dealing with overweight or obesity issues. Given that weight loss has been consistently shown to improve glycemic control in patients with T2D, as highlighted in previous studies [12-16,25], our findings reinforce the importance of weight management as an integral part of diabetes care. Self-monitoring can enhance patient awareness and engagement in their health management, leading to better outcomes. This aligns with the growing body of evidence suggesting that patient engagement and empowerment are critical in managing chronic conditions such as T2D [34,40,63,64].

Self-monitoring is the centerpiece of behavioral weight loss intervention programs. A significant association between self-monitoring and weight loss was consistently reported for various health conditions; however, the level of evidence was weak due to methodological limitations [65]. The use of self-monitoring in behavioral changes has a strong theoretical foundation. Self-management was defined as “the personal application of behavior-change tactics that produces a desired change in behavior” [66]. Through self-management interventions, individuals learn to identify occurrences of their own target responding, accurately self-recording the target response, self-evaluating their behavior, and self-delivering reinforcement as a consequence [67].

Although self-monitoring has been described as the cornerstone of behavioral treatment for weight loss, there is a limited examination conducted in the literature [65]. More recently, self-weighing has been introduced as a monitoring component. Daily weighing is valuable for individuals trying to lose weight or prevent weight gain [68]. Consistent with our findings, frequent self-weighing was associated with a lower fat intake, a greater history of dieting to lose weight, and a lower current BMI [68,69].

Previous systematic reviews provided extensive evidence that self-monitoring via digital health, including weight, diet, and physical activity, is associated with superior weight loss [36]. It was specifically shown how distinct features of a digital therapeutic app have the potential to deliver equitable person-centric care and how digital engagement can play a key role in enhancing a person’s chronic condition self-management [63,64,70,71].

Self-monitoring has been shown repeatedly to be an important feature of behavioral weight loss digital programs [71]. Self-monitoring of weight and diet were positively correlated with weight loss, and the more consistently the monitoring occurred, the better the weight loss [72,73]. Self-monitoring is also a core component of behavioral obesity treatment, but there is limited knowledge about the efficacy of digital self-monitoring of weight in diabetes [36]. We had previously demonstrated how digital engagement and digital blood pressure monitoring may improve diabetes management [34,74]. Prominently, in this study, the WM and NWM groups were not different in their digital engagement. In addition, the median household income distribution of users in both groups was comparable, suggesting that the digital solution is desired and affordable across lower-, middle-, and high-income levels to enhance glycemic and weight loss outcomes. Mobile apps can successfully help patients lose weight and represent a cost-effective and accessible alternative to intensive in-person weight loss programs [51].

From a psychological perspective, it is assumed that individuals using a digital platform may develop more active roles in managing their health, and self-monitoring affects health in part or in whole via the placebo effect, initiated by mindset modulations [75]. In the realm of physical exercise, a compelling body of research highlights the remarkable impact of mindset on various health parameters. It has been demonstrated that individuals’ mindsets about stress could profoundly alter their cortisol levels and influence various hormonal and cardiovascular functions when confronted with stressful situations [76]. Levy et al [77] conducted a noteworthy study that revealed a significant association between individuals’ mindsets about aging and their cardiovascular function as well as their actual longevity. A notable example of this phenomenon is evident in the study conducted by Crum and Langer [28], who investigated the effects of mindset on hotel room attendants. These workers, upon adopting the mindset that their daily work constituted a form of beneficial exercise, experienced substantial improvements in several critical health indicators, including weight, BMI, and systolic blood pressure. The potential mechanism may include beliefs and feelings of control people have over their health [28,78]. Collectively, these studies illuminate the potent role of the mindset in shaping various aspects of physical health, providing a background for the potential effect of weight monitoring on BG levels through mindset changes, considering the absence of a quasi-causal association between BMI and BG levels. There is evidence supporting the idea that the placebo effect plays a role in prompting the psychological benefits associated with health-related outcomes [79]. Treatments are delivered in a context that includes social and physical signals, verbal suggestions, and clinical history. This context is actively interpreted by the brain and can elicit expectations, memories, and emotions, which in turn can influence health-related outcomes in the body [79]. Considering the absence of a quasi-causal association between BMI and BG levels, one may consider the effect of self-weighing on BG levels to be mediated by perceptional processes, including mindset modulation.

In agreement with previous studies, we found that the WM group, which monitored their weight, also improved their BMI levels over time [80]. There is strong and consistent evidence that obesity management can delay the progression from prediabetes to T2D and is highly beneficial in treating T2D [80-83]. A significant overlap between T2D and overweight or obesity in etiology and disease mechanisms was broadly investigated. Previous studies have shown a significant improvement in all diabetes-related outcomes, including weight reduction, in patients with T2D and those who have overweight [17]. Controlling both diseases through weight management requires an intensive multidisciplinary approach [84,85]. As body weight increases, patients become more insulin resistant [86], which further drives the need for higher doses of antihyperglycemic medications to keep BG levels in the target range, which then may increase weight gain [17]. It was observed that in obese individuals, adipose tissue releases increased amounts of nonesterified fatty acids, glycerol, hormones, proinflammatory cytokines, and other factors that are involved in the development of insulin resistance. Insulin resistance associated with dysfunction of pancreatic islet beta cells results in the failure to maintain BG levels within the target range [86]. Losing weight can alleviate many of these issues. While losing weight, the pancreas is better able to keep up with the body’s need for insulin. In some cases, weight loss is enough to restore BG to a normal level, which eliminates diabetes or even lowers the need for insulin therapy or other medications to control diabetes [87]. However, other important components may also play a role in weight and BG levels, and other laboratory tests may need to be performed by health care providers.

Monitoring several chronic conditions may have the potential to offer a greater means for helping people with diabetes who have overweight or obesity effectively modulate their glycemia and weight than managing each condition separately. The findings of this study suggest the need for further exploration of how digital health platforms can be effectively integrated into routine clinical practice. Future research should focus on how these tools can be optimized for individual patient needs and how they can be incorporated into broader diabetes management programs. In addition, exploring the long-term impacts of such interventions on patient outcomes and health care use will be valuable. We expect that our analytical framework will be useful for examining other chronic conditions and metabolic syndrome outcomes (eg, lipid profile).

### Limitations

As in all studies involving retrospective real-world data, groups were not randomly assigned, and treatment protocols were not prescribed. The propensity score matching approach, while comprehensive, introduces potential biases due to selection methods, which may not be fully mitigated. Unobserved confounders, measurement errors, and other limitations inherent to observational studies may still impact the validity of causal inferences. Nevertheless, propensity score matching represents a rigorous analytical technique that is widely accepted and applied in observational studies to approximate the design and control of randomized controlled trials, making it a valuable tool in the pursuit of causal inference. In this study, we relied on self-reported data for measurements, including weight and BMI. Although self-reporting is a common and practical method in many observational and digital health studies, it can be subject to inaccuracies due to factors such as recall bias or the desire to present oneself in a certain light.

In this real-world data analysis, we designed our timescale to capture changes over a 6-month period both before and after the initiation of weight monitoring. Nevertheless, it is worth noting that the research question of interest in this study could potentially be explored at various temporal scales, including daily, weekly, or monthly intervals. Given the practical challenges associated with monitoring daily changes in real-world settings, most studies in this domain tend to emphasize monthly fluctuations. Moreover, while BG levels offer real-time data and are sensitive to immediate changes in diabetes management, they do not provide a comprehensive view of long-term glycemic control. In addition, longer-term weight monitoring can provide a long-term perspective on glycemic control. Monitoring HbA_1c_ levels over a longer period would provide additional insights into the long-term effects of self-monitoring of weight on glycemic control. However, our study was designed to assess the short-term impacts of digital self-monitoring of weight on BG levels with increased resolution compared to HbA_1c_. The 6-month period was chosen as it provides a sufficient window to observe significant changes in BG levels in response to weight management, without extending to long-term effects where other variables might confound the results. In addition, the average BMI of the participants was 35.0 (SD 7.3), which is considered a unique population of obesity. Furthermore, longer-term follow-up with these participants is needed because it is not clear how long this weight loss and glycemic improvement will last and what needs to be added for increased sustainability.

### Conclusions

In summary, our study underscored the tangible benefits of self-monitoring of weight in the modulation of BG levels among people with diabetes. By leveraging an innovative analytical framework, we found that self-monitoring of weight led to significant reductions in BG levels in the WM group, despite the lack of a direct causal link between BMI fluctuations and BG changes. Drawing from extensive evidence, both historical and from our study, the act of self-monitoring seems to foster a heightened sense of agency and potentially influence health outcomes through mindset modulations.

From a practical standpoint, these findings reinforce the importance of digital health tools in chronic disease management, especially in the realms of diabetes and obesity. Digital self-monitoring platforms not only offer scalable and affordable solutions but also empower individuals to take a proactive role in their health journey. Moreover, the convergence of digital health tools with robust psychological mechanisms, such as the placebo effect and mindset modulation, paves the way for a holistic approach to health care.

This research may also open the door to a myriad of possibilities. While we have illuminated the potential effects of self-monitoring of weight on diabetes management, similar methods could be deployed to investigate the impact on other chronic conditions and metabolic syndrome outcomes. Furthermore, with advancing technology, more granular, real-time data can be leveraged to delve deeper into the daily or even hourly impacts of such interventions. Future studies should also focus on investigating the mechanisms underlying the comorbidity of diabetes and obesity and their management, identifying, and applying mediation models that drive behavioral change that goes beyond multiple chronic conditions.

In light of the significant overlap between T2D and obesity, there is an imperative need to conceptualize and design multifaceted interventions. Blending digital innovation, behavioral science, and clinical knowledge, we can usher in a new era of person-centric health care that is not only responsive but also preemptive. The journey has just begun, and the road ahead promises transformative potential for patients and health care systems alike.

## Abbreviations

BG: blood glucose
NWM: non–weight monitoring (group)
T2D: type 2 diabetes
WM: weight monitoring (group)
